# Evaluating the Strange Situation Procedure (SSP) to Assess the Bond between Dogs and Humans

**DOI:** 10.1371/journal.pone.0056938

**Published:** 2013-02-20

**Authors:** Therese Rehn, Ragen T. S. McGowan, Linda J. Keeling

**Affiliations:** 1 Department of Animal Environment and Health, Swedish University of Agricultural Sciences, Uppsala, Sweden; 2 Department of Animal Environment and Health, Swedish University of Agricultural Sciences, Uppsala, Sweden; Université Pierre et Marie Curie, France

## Abstract

The Strange Situation Procedure (SSP) is increasingly being used to study attachment between dogs and humans. It has been developed from the Ainsworth Strange Situation Procedure, which is used extensively to investigate attachment between children and their parents. In this experiment, 12 female beagle dogs were tested in two treatments to identify possible order effects in the test, a potential weakness in the SSP. In one treatment (FS), dogs participated together with a ‘familiar person’ and a ‘stranger’. In a control treatment (SS), the same dogs participated together with two unfamiliar people, ‘stranger A’ and ‘stranger B’. Comparisons were made between episodes within as well as between treatments. As predicted in FS, dogs explored more in the presence of the familiar person than the stranger. Importantly, they also explored more in the presence of stranger A (who appeared in the same order as the familiar person and followed the same procedure) than stranger B in SS. Furthermore, comparisons between treatments, where a familiar person was present in FS and stranger A was present in SS, showed no differences in exploration. In combination, these results indicate that the effect of a familiar person on dogs' exploratory behaviour, a key feature when assessing secure attachment styles, could not be tested reliably due to the order in which the familiar person and the stranger appear. It is proposed that in the future only counterbalanced versions of the SSP are used. Alternatively, since dogs reliably initiated more contact with the familiar person compared to the strangers, it is suggested that future studies on attachment in dogs towards humans should focus either on the behaviour of the dog in those episodes of the SSP when the person returns, or on reunion behaviour in other studies, specially designed to address dog-human interactions at this time.

## Introduction

For decades, the Ainsworth's Strange Situation Procedure (ASSP) has been used extensively to investigate attachment between children and their parents (e.g. [1]–[4]) and lately, modified versions of the test have been used to assess attachment in dogs. However, there has been some criticism of this methodology, due to possible order effects [5]–[6]. This paper briefly summarises the theory behind the original ASSP from the human psychology literature and describes how the test has been modified in the anthrozoology literature, where it is commonly known as the Strange Situation Procedure (SSP). It reports on an experiment specifically designed to evaluate potential problems of order effects in the SSP when it is used to investigate attachment between dogs and humans. Lastly, based on the results from this study with experimental dogs, we make recommendations to improve the reliability of future versions of the SSP and propose alternative ways to investigate attachment styles between dogs and their owners.

Attachment refers to a particular type of long-lasting affectional bond that develops between two individuals (e.g. [1], [7], [8]) and functions to facilitate reproduction, provide a sense of security and reduce feelings of stress and anxiety [9]. According to attachment theory [7], [10], the attached individual shows a preference for the attachment figure and gets distressed when involuntarily separated from it. At least three different attachment styles have been described in human psychology [11]: *secure* (individual uses the attachment figure as a secure base and voluntarily moves away to engage in exploration/play and where reunion behaviour is characterized as smooth and positive), *ambivalent/resistant* (individual is distressed when separated from the attachment figure, but resists comfort when reunited) and *avoidant* (individual shows no signs of distress when separated from the attachment figure and explores/plays regardless of whether the attachment figure is present or not).

The ASSP was developed [1] to investigate the variety of attachment styles between young children (<2 years old) and their primary caregiver (usually their mother). It includes mildly challenging situations and aims to activate the innate, biological ‘attachment system’ to observe whether the attached individual discriminates between the attachment figure and an unfamiliar person by seeking proximity to the attachment figure and if this proximity seeking behaviour is especially pronounced when the subject is feeling anxious (e.g. when separated from the attachment figure). In contrast, when the attachment figure is present, the subject may use this person as a secure base from which to explore the environment. Reunion behaviour (greeting) between attached individuals is proposed to be the most relevant feature of the ASSP to assess the style of attachment [11].

Because the dog-owner relationship has been proposed to resemble the child-parent bond [12]–[15] modified versions of the ASSP, from here on referred to as the SSP, have been used to study attachment behaviour in dogs (e.g. 6,13,15–18). Overall, studies show that pet dogs react to the SSP in a similar way as infants when participating together with their owners, indicating that there is an attachment bond present between them. The categorization of dogs into different attachment styles has so far been more or less neglected (exceptions: [13], [15]). It is interesting to note though that while behaviour during reunion is considered to be the most important aspect of the ASSP to assess attachment styles in humans, this aspect is not the main focus in the SSP used to assess attachment between dogs and their owners. Instead, identification of a secure attachment style has been the focus, assessed mainly by levels of exploration and play [6], [18].

However, in dogs, the level of exploration of the novel environment quickly decreases during the test [6], [13], [16], [18] and there is some evidence of the same pattern regarding play behaviour [6]. This makes interpretation of these behaviours sensitive to any order effects in the test. Some examples of order effects are that the owner is the one accompanying the dog from the start of the test and the reaction of the dog to the return of a person varies depending on whether the dog was alone or not in the preceding episode. To overcome these possible order effects, [18] used a counterbalanced version of the test and showed that dogs seemed to use their owner as a secure base, e.g. they explored more and performed more individual play in the presence of the owner.

In contrast to [18], who balanced for possible order effects to allow them to investigate indicators of secure base effects, the aim of this study was to focus directly on investigating these order effects. To examine this, we chose to add a treatment with two strangers and to use a cross-over design. Thus, we built upon the approach in [18], but the main difference in our approach, and what has not been done previously, is to include a ‘control’ treatment where no familiar person is ever present with the dog in the room. In one treatment (FS), the dog participated in the SSP together with a familiar person (F) and a stranger (S). In the other treatment (SS), the dog participated together with two unfamiliar people, stranger A (S_A_) and stranger B (S_B_). This allowed for comparisons within as well as between treatments to investigate the effect of the familiar person on the dogs' reactions during the test, i.e. whether the familiarity of the person or order of appearance of a person as well as the mere sequence of events impacted the dogs' responses. Attached individuals should demonstrate a selective response to the attachment figure (F), different from behaviours shown to a non-attachment figure (S, S_A_ and S_B_). Using the dogs at our university enabled us to use people experienced with research as both the familiar and unfamiliar person to further standardize the experiment. Laboratory dogs, housed at a research facility contribute to a more controlled experiment in the sense that uniform groups matched for breed, age, previous experience, housing and kinship can be used. Companion dogs probably have a different relationship with their owner than do research dogs with their handler. But the aim of this study was not to assess different features of the dog-owner bond, but to investigate methodological issues of a version of the SSP, using each dog as its own control, with minimised confounding effects.

We chose the protocol used in [18], where the reunion with the owner occurs immediately after the dog has been alone in the room. This was to be able to include a ‘pure’ reunion between the dog and the familiar person because reunion between children and their parents has been suggested to be an important tool when assessing attachment, but this has been less investigated in dog literature.

## Materials and Methods

### Ethics statement

This study was carried out in strict accordance to the protocol approved by the Swedish Ethical Committee on animal research in Uppsala, Sweden (Permit Number: C130/8). With regards to the protocol for the participating humans in this study, no ethical approval was required according to Swedish legislation (Ethical review act, 2003:460).

### Subjects

Twelve intact female beagle dogs (aged 25 months (±0.6 (SE)), kept at the Swedish University of Agricultural Sciences, were included. Before the study, dogs were used for behaviour studies on human-animal interactions and positive affective states in dogs (e.g. [19], [20]). They had never participated in studies where they were exposed to negative treatment or invasive measures. The dogs were housed in stable groups of 3 dogs indoors (24.3 m^2^) and in larger groups (approximately 6 dogs/group) outdoors (220–330 m^2^) during the day between 8:00 and 15:30. They were individually fed indoors, at around 7:30 and at 16:00. The dogs were walked regularly around the campus by their caretaker. All dogs were used to wearing a heart rate (HR) monitor (Polar® Vantage S810) from previous studies.

All dogs participated in two different treatments, FS (familiar person, stranger) and SS (stranger A, stranger B) with one week between tests. Six dogs started with treatment FS and the other 6 with treatment SS. The kinship between dogs was taken into consideration (there were 4 pairs of full sisters in the group and sisters were allocated to start with different treatments). Six of the dogs always performed the test in the morning and the other 6 dogs in the afternoon, balanced across treatments.

The people acting as the familiar persons (F) in treatment FS were 2 females who had been working with the dogs for nearly 2 years prior to this experiment, e.g. they spent most of the day together with the dogs for several weeks when they arrived as naïve research dogs to the facility and they had participated with the dogs in previous studies on positive dog-human interactions. They were each assigned to 6 dogs. A third female acted as the stranger (S) in treatment FS and 2 other females were allocated the roles of strangers (S_A_ and S_B_, respectively) in treatment SS. In the SS treatment, S_A_ appeared in the same order and followed the same procedure as the person playing the F role in the FS treatment, and S_B_ acted equivalent to the person playing the S role in treatment FS. The persons acting as strangers had never met the dogs prior to the experiment.

### Test area

**Figure 1:**
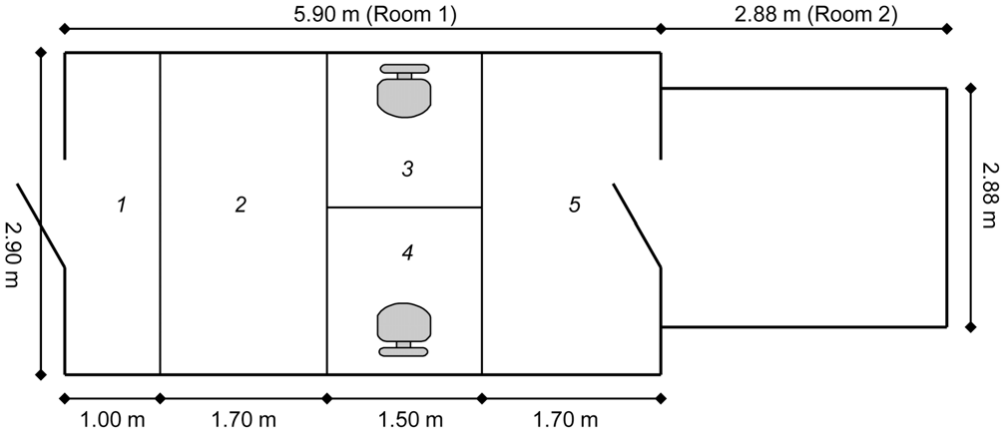
Overview of the test area. The black lines represent the tape on the floor that divided the larger room (room 1) into five zones (1: Near entrance door, 2: Neutral zone, 3 and 4: familiar person's/S_A_'s zone and stranger's/S_B_'s zone each containing a chair, 5: Neutral zone containing a rope tug-toy). The entrance door is at the left hand side of the figure and there is a door between room 1 and room 2 shown to the right in the picture. Room 2 was empty and was available to the dogs from episode 4.

The test area (Figure 1) comprised two relatively bare rooms (room 1: 17.1 m^2^ and room 2: 8.4 m^2^) separated by a door closed at the beginning of each test. In room 1, five areas were marked on the floor with tape to facilitate monitoring of the dogs' location during the test. Three video cameras covered the whole area of room 1: one digital video camera (SONY HDR-SR10E) and two wireless surveillance cameras (VIVOTEK network camera, PT3124). The rooms were cleaned and disinfected between tests. The dogs had no previous experience of the rooms before participating in the experiment.

### Procedure

Before the experiment, people involved were informed about how to act during the test and had practiced the procedure (Table 1). They were instructed, unless required otherwise in the procedure, to interact with the dog only if it was within one arm's length distance and focused on them for more than 2 s, or if the dog initiated physical contact. If the dog initiated contact in this way, the person was allowed to stroke the dog once. Within the restrictions of the protocol, people were requested to act as naturally as possible.

**Table 1:** Episode description.

Episode	Minute	FS	SS	Main event in each episode
1	1^st^-3^rd^	F	S_A_	F/S_A_ sits quietly in the chair completing a crossword (ignores the dog).
2	4^th^–6^th^	F+S	S_A_+S_B_	S/S_B_ enters, sits quietly in chair with crossword for 1 min, starts a conversation with F/S_A_ for the second min, then sits on floor and initiates play with the dog using the rope during the last min. Returns to chair after 45 sec if dog does not want to play. F/S_A_ leaves the room unobtrusively at the end of the episode.
3	7^th^–9^th^	S	S_B_	S/S_B_ continues play/initiates play again with the dog. Returns to chair after 45 sec if dog does not want to play. 20 sec before end of episode, S/S_B_ opens the door to room 2 and then leaves via the entrance door in room 1.
4	10^th^–12^th^	Alone	Alone	Dog is alone in test room, with access to room 2.
5	13^th^–15^th^	F	S_A_	F/S_A_ enters, waits 7 sec, greets the dog for 10 sec, then sits down with crossword and ignores the dog.
6	16^th^–18^th^	S	S_B_	S/S_B_ enters, waits 7 sec, greets the dog for 10 sec, then sits down with crossword and ignores the dog. F/S_A_ leaves the room when S/S_B_ stops greeting the dog.

Episode overview of the protocol used in treatments FS and SS where the dog, a familiar person (F) and a stranger (S) participated in treatment FS and the dog, stranger A (S_A_) and stranger B (S_B_) participated in treatment SS.

The protocol of the SSP was based on condition A in [18]. The SSP took 18 min and consisted of 6 episodes each lasting for 3 min. To maintain potential interest for exploring, access to room 2 was only available for the dogs from episode 4 onwards. A familiar person collected the experimental dog from the kennel and walked to the test area, situated in a building approximately 50 m from the kennels. The HR monitor was placed around the chest of the dog and then, depending on whether it was an FS or an SS treatment, the familiar person/stranger A entered the test room with the dog. She closed the entrance door behind her and walked to the middle of the room where the dog was released. The familiar person/stranger A then sat down in her allocated chair (which chair/side of the room was balanced equally between dogs to avoid potential location preference, but each individual dog had the familiar person/stranger A on the same side of the room in both treatments).

### Data collection

The behaviour, location and cardiac activity of the dogs were recorded during all episodes. The video footage was analyzed by two trained observers (each observer analyzed half of the videos from each treatment). All behaviours, as well as the location of the dogs, were recorded when it was in room 1, using instantaneous sampling every 5 s, one/zero sampling and continuous recording (Appendix S1). When the dog was in room 2, only the amount of time spent in the room was recorded. HR data were registered as mean HR every 5 s and stored in a wrist watch receiver synchronized with the collection of behavioural data to better interpret HR data in relation to physical activity. Data were then transferred into the Polar® Precision Performance software (version 4.0) to be exported for further analyses.

### Comparisons and hypotheses

It is standard in the SSP to compare episodes where the familiar person and/or the stranger are present in the room in order to investigate secure base and proximity seeking behaviour. However, since S_A_ in the SS treatment interacted with the dog in the same way and in the same order as F did in the FS treatment, comparisons between treatments were also possible. This allowed investigation of whether it was the previous experience of the dog with the familiar person prior to the study that was critical or just that they were the first person with the dog in the new environment.

**Figure 2:**
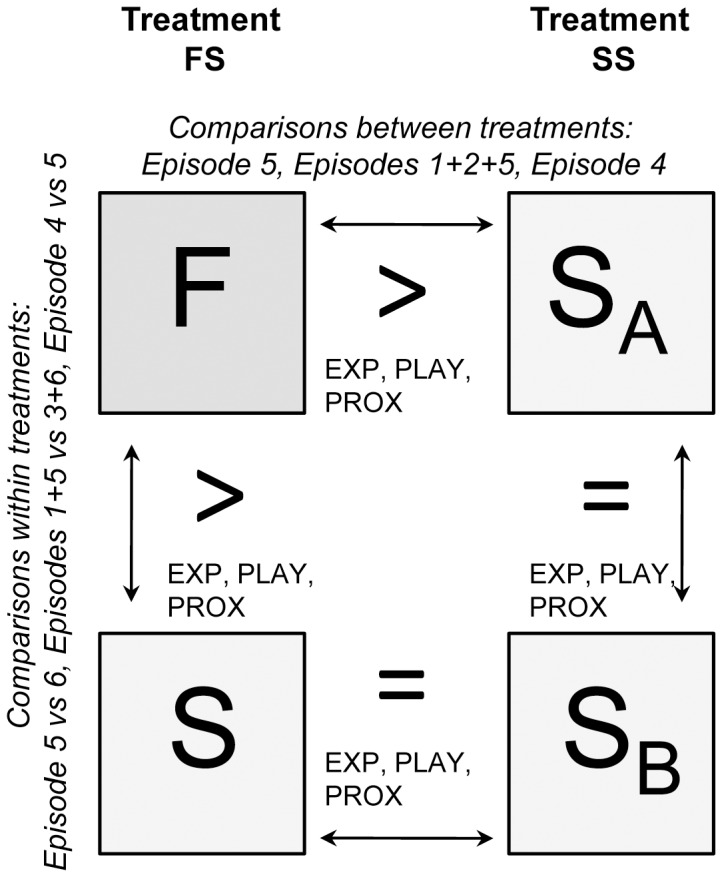
A schematic representation of predictions. Closed boxes refer to episodes where either F (familiar person) or S (stranger) was present in the room in the FS (familiar stranger) treatment or S_A_ (stranger A) or S_B_ (stranger B) was present in the room in the SS (stranger stranger) treatment. The arrows indicate the comparisons that were made. According to our hypothesis, if the ASSP is a reliable method to use when assessing the bond between dogs and humans, dogs should *explore* (EXP) and *play* (PLAY) more in the presence of the familiar person and they should show more *proximity seeking behaviours* (PROX) towards the familiar person.

Within treatment comparisons were made between episodes where F (treatment FS) or S_A_ (treatment SS) was present and episodes where S (treatment FS) or S_B_ (treatment SS) was present. It was expected that dogs would show more exploratory and play behaviour when with F compared to when with S in the FS treatment (Figure 2). This difference was not expected between the episodes where S_A_ was present and when S_B_ was present in the SS treatment, allowing the exclusion of order effects in the SSP. The amount of proximity-seeking behaviour was expected to be higher when F was/had been absent compared to when S was/had been absent in the FS treatment. These differences were not expected between S_A_ and S_B_ in treatment SS.

Between treatment comparisons were made between episodes where F was present in treatment FS and episodes where S_A_ was present in treatment SS. We predicted that these comparisons would reveal that dogs explore and play more, as well as show more proximity seeking behaviour in the presence of F in treatment FS than in the presence of S_A_ in treatment SS.

To investigate greeting behaviour towards the different people, within treatment comparisons were based on data from the 1^st^ minute of episode 5 (min 13, F or S_A_ present) and the 1^st^ minute of episode 6 (min 16, S or S_B_ present). Between treatment comparisons of greeting behaviour towards F and S_A_ were made based on data from the 1^st^ minute of episode 5 (min 13) in FS and SS respectively. We expected that dogs would initiate more physical contact with F during reunion, as well as show higher levels of those behaviours related to social interactions, such as tail wagging, lip licking and body shaking compared to when reunited with any of S, S_A_ or S_B_.

Assuming no difference in physical activity, the HR of the dogs was expected to be lower in the company of F compared to S in treatment FS, but no such difference was expected when the dog was with either S_A_ or S_B_ in treatment SS.

### Statistical analyses

Behavioural data recorded instantaneously were summarised as the mean proportion of sample points per minute or per episode(s) and dog. Behaviours recorded continuously were summarised as mean frequency per minute or per episode(s) and dog. All statistical tests for behavioural differences were performed using non-parametric tests (Wilcoxon signed rank). HR data were summarised as mean HR per minute or per episode(s) and dog, then analyzed using Mixed models where minute or episode were considered as fixed effects and dog was included as a random effect. The tests were executed in SAS® (version 9.2). Inter-observer agreement for observations of behaviour from the videos was tested using the attribute agreement analysis in Minitab® (version 16).

## Results

Approximately 17% of the video recorded material was analysed by both observers and inter-observer agreement was always above 86%. Only 4 dogs played with the stranger when invited to do so in episode 2 and 3, and at a very low frequency (33 observations out of the total possible 576 sample points, evenly distributed across treatments). Hence, social play was not included in our further analyses. In the remainder of the paper, play refers to individual play (dog is carrying/throwing and/or chewing on toy/water bowl/leash).

Results are reported in four different sections related to what was investigated; exploration and play behaviour, proximity seeking behaviour, greeting behaviour and cardiac activity. Each section consists of two sub-sections describing results from the within and the between treatment comparisons, respectively.

### Exploration and play

Levels of exploration and play were analysed to investigate possible secure base effects of the accompanying person. Also, the amount of time spent in room 2, to which the dogs had access from episode 4 onwards was considered as ‘exploratory behaviour’ and was related to these secure base effects. These measures have been the main focus in previous attachment studies in dogs in order to investigate secure base, although they do not cover the whole aspect of secure attachment according to human literature.

#### Comparisons within treatments (episode 5 vs. 6, episode 1+5 vs. 3+6)

**Figure 3:**
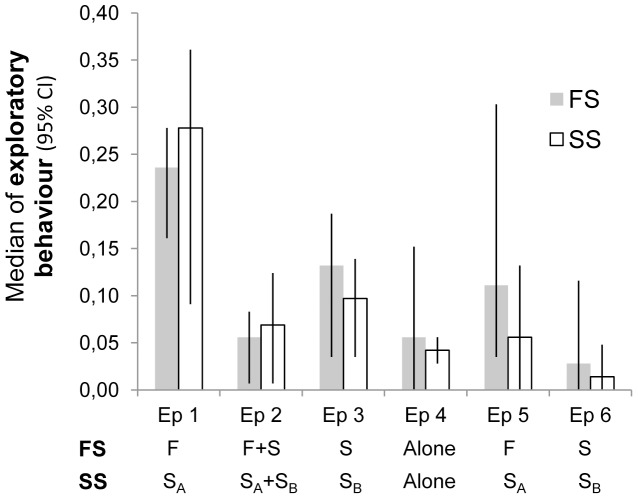
Exploration levels in both treatments during the test. Level of exploration (median proportion of sample points/episode presented together with 95% confidence intervals) across the whole test procedure (episode (Ep) 1–6) in treatment FS and treatment SS. F = familiar person, S = stranger, S_A_ = stranger A and S_B_ = stranger B.

Comparison within treatment FS showed that dogs spent more time exploring (Wilcoxon signed rank: *T* = 18, *P* = 0.03) in episode 5, when the familiar person was present compared to episode 6, when the dog was accompanied by a stranger (Figure 3). Even in treatment SS, dogs spent more time exploring (*T* = 12, *P* = 0.05) in episode 5, with stranger A present, than in episode 6, with stranger B present, although this difference was not as great as in the FS treatment. The level of individual play or the time spent in room 2 did not differ between episode 5 and episode 6 within either treatment.

Also, comparisons between the combined episodes where only the familiar person was present *vs.* the episodes where only the stranger was present in the FS treatment showed that dogs explored more (*T* = 39, *P*<0.001) in the presence of the familiar person (episode 1+5: 0.18 (0.15; 0.25) (median proportion of sample points (lower; upper 95% confidence interval)); episode 3+6: 0.08 (0.03; 0.15)). When comparing the equivalent episodes within the SS treatment, the same pattern was found (episode 1+5: 0.15 (0.06; 0.24); episode 3+6: 0.06 (0.03; 0.09); *T* = 26.5, *P* = 0.004), i.e. dogs explored more in the presence of stranger A than in the presence of stranger B. When combining the episodes, no differences in play behaviour or the time spent in room 2 were found within either treatment.

#### Comparisons between treatments (episode 5, episode 1+2+5)

Comparisons of exploratory behaviour during episode 5, where a familiar person was present in the FS treatment and where stranger A was present in the SS treatment, showed no differences (Fig. 3), but dogs played more (*T* = 16.5, *P* = 0.02) in the company of a familiar person (0.08 (0.00; 0.28) than when with stranger A (0.01 (0.00; 0.08)). There were no differences in the time spent in room 2 when comparing episode 5 (familiar person present in FS, stranger A present in SS) between treatments.

Comparisons between all those episodes in the FS treatment where the familiar person was present (episode 1+2+5) and the same episodes where stranger A was present in treatment SS, showed no overall difference between treatments regarding exploratory behaviour. Again, however, it was found that dogs showed more play behaviour (*T* = 21, *P* = 0.01) in the presence of the familiar person in the FS treatment (0.03 (0.002; 0.15) than they did in the company of stranger A in the SS treatment (0.005 (0.00; 0.04).

### Proximity seeking behaviours

To investigate the dogs' proximity seeking behaviour in the test, as an indicator of their attachment to the accompanying person, levels of physical contact with and orientation towards the person were measured, as well as the location of the dog (within the person's zone or not). When the person was absent, the levels of contact with the person's empty chair and contact with or orientation towards the entrance door were measured, as well as the dog's location (near entrance door).

#### Comparisons within treatments (episode 5 vs. 6, episode 1+5 vs. 3+6, episode 4 vs. 5)

**Figure 4:**
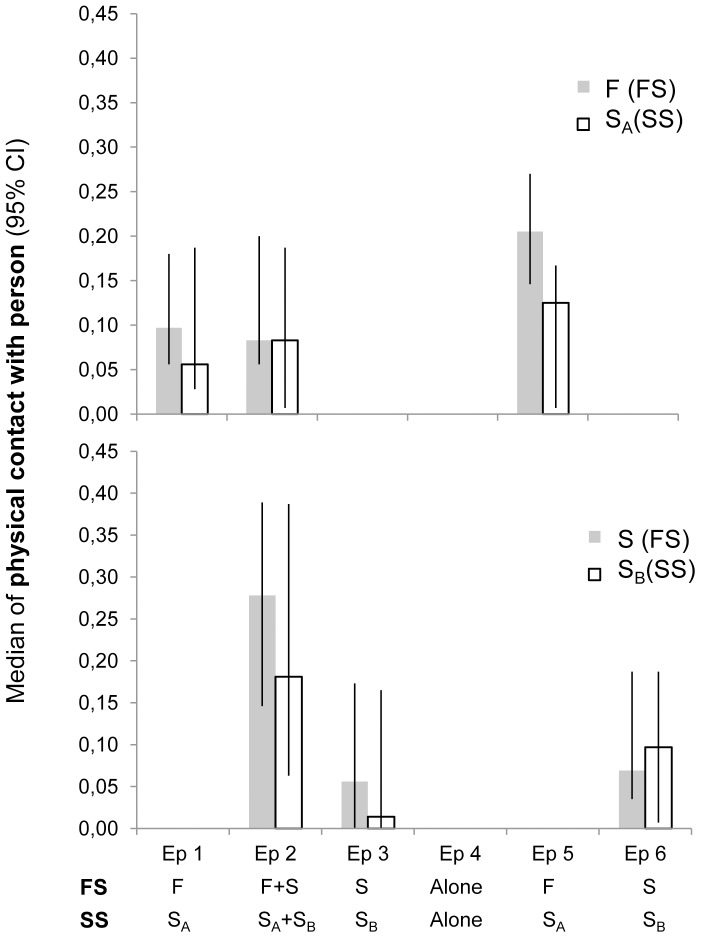
The level of physical contact with humans initiated by dog in both treatments. Time spent in physical contact with person (median proportion of sample points/episode presented together with 95% confidence intervals) during the whole test procedure (episodes (Ep) 1–6) in treatment FS and in treatment SS. F = familiar person, S = stranger, S_A_ = stranger A and S_B_ = stranger B.

In treatment FS, dogs initiated physical contact more often with the familiar person (*T* = 37.5, *P* = 0.002) in episode 5, than they did with the stranger in episode 6 (Figure 4). No other differences in proximity seeking behaviours were found within treatment FS. In the SS treatment, no differences in the level of physical contact or other proximity seeking behaviours were found between episode 5 (stranger A present) and episode 6 (stranger B present).

Comparisons between the two episodes where only the familiar person was present in the FS treatment (episodes 1+5) *vs.* the two episodes where only the stranger was present in the room (episodes 3+6) confirmed that dogs initiated more physical contact (*T* = 32.5, *P* = 0.007) with the familiar person (0.17 (0.09; 0.18)) than with the stranger (0.08 (0.03; 0.13)). In treatment SS, dogs did not differ in their proximity seeking behaviours towards either stranger.

During episode 4 (dog alone), dogs were more oriented towards the entrance door compared to episode 5 (familiar person or stranger A present) in both treatments (FS: *T* = 30, *P* = 0.005; SS: *T* = 28, *P* = 0.01) (Table 2). They were also more often located near the door when they were alone in both treatments (FS: *T* = 32, *P* = 0.01; SS: *T* = 39, *P* = 0.0005) compared to when accompanied by the familiar person (FS) or stranger A (SS). No other proximity seeking behaviours differed between episode 4 and episode 5 in either treatment.

**Table 2:** Proximity seeking behaviours.

	Oriented towards door		Located near door	
Episode	FS	SS	FS	SS
4	0.45 (0.15; 0.64)	0.36 (0.15; 0.62)	0.51 (0.18; 0.68)	0.54 (0.29; 0.66)
5	0.01 (0.00; 0.09)	0.04 (0.00; 0.24)	0.10 (0.06; 0.21)	0.19 (0.06; 0.27)

The median proportion of sample points/episode (lower; upper 95% confidence interval) of dogs being oriented towards the door and located near door during episodes 4 (dog alone) and 5 (familiar person present (in treatment FS) or stranger A present (in treatment SS)).

#### Comparisons between treatments (episode 5, episode 1+2+5, episode 4)

In episode 5, dogs spent more time in physical contact (*T* = 31, *P* = 0.01) with the familiar person in treatment FS than they did with stranger A in the SS treatment (Fig. 4).

When comparing all episodes when the familiar person was present in the FS treatment with all episodes when stranger A was present in the SS treatment (episodes 1+2+5), dogs initiated more physical contact (*T* = 27.5, *P* = 0.03) with the familiar person (0.14 (0.08; 0.19)) than they did with stranger A (0.08 (0.03; 0.17)). There were no other differences in proximity seeking behaviour when all episodes where the familiar person was present were combined and compared with all episodes where stranger A was present.

No differences in proximity seeking behaviours during episode 4 (dog alone) were found between treatments.

### Greeting behaviour

Since greeting behaviour is considered an important measure in the evaluation of attachment between humans, the initial response of the dog upon reunion with the different people was compared within treatments (min 13 *vs.* min 16) and between treatments (min 13). The amount of physical contact initiated by the dogs as well as behaviours commonly observed in social contexts (tail wagging, lip licking, body shaking and vocalising) are reported below.

#### Comparisons within treatments (min 13 vs. min 16)

Dogs initiated a higher level of physical contact (*T* = 27.5, *P* = 0.002) with the familiar person (0.54 (0.36; 0.73)) compared to the stranger (0.21 (0.04; 0.46)) in treatment FS. Also, dogs performed a higher frequency of lip licking (*T* = 30, *P* = 0.005) when greeting the familiar person (0.38 (0.17; 0.81)) than when greeting the stranger (0.17 (0.03; 0.25)). There were no significant differences in the levels of body shaking, tail wagging or vocalising when greeting the familiar person *vs*. the stranger. In the SS treatment, there was no difference regarding the level of physical contact, body shaking or vocalising when greeting either stranger A or stranger B. However, dogs performed more lip licking (*T* = 24, *P* = 0.01) when reunited with stranger A (0.25 (0.18; 0.48)) than with stranger B (0.17 (0.00; 0.31)).

#### Comparisons between treatments (min 13)

During the first minute of episode 5, comparisons showed that dogs initiated more physical contact (*T* = 34.5, *P* = 0.004) when reunited with the familiar person (0.54 (0.36; 0.73)) in treatment FS than when reunited with stranger A (0.29 (0.02; 0.50)) in treatment SS. Although not significant, dogs tended to vocalise more (*T* = 7.5, *P* = 0.06), show more tail wagging (*T* = 16, *P* = 0.06) and more body shaking (*T* = 19.5, *P* = 0.06) when the familiar person returned in treatment FS compared to when stranger A came back in treatment SS.

### Heart rate and physical activity

To better interpret HR measures in relation to physical activity, main behaviours (lying, sitting, standing, walking and running) were also investigated and presented in this section.

#### Comparisons within treatments (episode 5 vs. 6, episode 1+5 vs. 3+6)

No differences in mean HR or in any of the main behaviours were observed within either treatment when comparing episode 5 with episode 6.

However when episodes within each treatment were combined, dogs had a higher mean HR (*F* = 4.65, d.f = 1, *P* = 0.05) during the two episodes when only the familiar person was present in the room (episode 1+5: 143.8 (137.7; 149.8) (mean beats/min (lower; upper 95% confidence interval of the mean))) compared to those episodes when only the stranger was present (episode 3+6: 138.6 (130.2; 146.9)) in treatment FS. Although, dogs were more physically active i.e. lying down less (*T* = 10.5, *P* = 0.03), when the familiar person was present (episode 1+5: 0.00 (0.00; 0.15) (median (lower; upper 95% confidence interval of median)), episode 3+6: 0.11 (0.00; 0.40)). In treatment SS, HR was higher (*F* = 10.58, *P* = 0.009) in episode 1+5 (143.3 (135.0; 151.6) compared to in episode 3+6 (134.2 (127.0; 141.3)), but in this treatment there were no differences regarding lying or any other main behaviour.

#### Comparisons between treatments (episode 5, episode 1+2+5)

No differences in HR or any other main behaviour that might reflect differences in physical activity were observed between treatments during episode 5. Neither did these variables differ between treatments when summarizing all episodes when the familiar person was present and comparing them with all episodes when stranger A was present.

## Discussion

The results indicate that there are order effects in this version of the SSP. Dogs explored as much in the presence of stranger A, who followed the same procedure as the familiar person, as they did in the presence of the familiar person. Since higher levels of exploration were observed in both the FS and the SS treatments there is no evidence that the familiar person affected the levels of exploration, but rather that it was the person who entered the room first with the dog that was important. Dogs did however initiate more physical contact with the familiar person, indicating that dogs preferred to be closer to the familiar person than a stranger, which is another important feature of attachment theory in humans. This aspect has, however, rarely been addressed in dog-human attachment studies.

In the sections below, each feature of attachment theory is described separately in relation to our results, to findings from previous studies on the dog-human relationship and to experiences from human psychology. This is followed by a general evaluation of the SSP, addressing particular methodological aspects. Lastly, we propose some future directions for studies of the dog-human attachment bond.

### Exploration and play

According to attachment theory [7], [10], attached individuals try to maintain proximity to each other and become distressed if separated involuntarily. One of the key features of *secure* attachment is that the attached individual should be more confident when accompanied by the attachment figure and move away from this secure base more often to engage in exploratory behaviour and play. In this current study, dogs explored more in the presence of the familiar person in the FS treatment compared to when they were with the stranger, as predicted. Nevertheless, they also explored more when accompanied by stranger A compared to stranger B in the SS treatment. When comparing across treatments, there was no difference in exploratory behaviour between episodes where the familiar person was present in FS compared to episodes where stranger A was present in SS. This implies that there was an effect of the sequence of events as dogs showed more exploration initially irrespective of who the accompanying person was, as well as upon reunion with the person who they first had entered with into the novel room. This confirms the speculation in other studies that the SSP is sensitive to order effects in the protocol [6], [13], [16], [18].

In contrast to our study, [18] found support for the owner acting as a secure base, mainly based on the mean levels of exploration in episode 1, where the dogs were accompanied by their owner in one condition, compared to the equivalent episode in the counterbalanced condition where dogs were accompanied by a stranger. Nevertheless, in line with our results, [18] found that within each treatment, exploration increased when either the owner or the stranger returned in episode 5. This suggests an effect on exploratory behaviour of reunion with a human *per se* after the dog has been alone, regardless of the relationship to the returning person.

There is little evidence to support that it is sufficient to meet the dog outside and enter the novel environment together with it for this person to act as a secure base for the dog during exploratory behaviour [21]. Rather, it seems as though the level of exploration is influenced by the time into and previous experiences in the test. Thus this version of the SSP does not seem to be able to demonstrate whether or not the familiar person acts as a secure base to increase exploratory behaviour in the dog. Therefore the order of the episodes should be taken into consideration when selecting the version(s) of SSP to use in future studies.

Contrary to studies on privately owned dogs, our dogs (almost) never engaged in social play when invited to do so. Unfortunately, this made it impossible for us to investigate the effect of the presence of a familiar person in relation to social play with a stranger, which is another measure of a secure base. When invited to play with the stranger, dogs in this study approached and initiated physical contact with the strangers instead of engaging in play. Although very socialized to people, these research dogs are probably not played with by their caretaker as often as typical companion dogs.

Individual play was expressed by all but three dogs in both treatments, but at low rates and results did not indicate any effect of a familiar person *within* treatment FS. Low levels of individual play have also been report in a study including pet dogs [16]. When comparing between treatments, the presence of a familiar person did increase the overall level of individual play behaviour, a finding which is consistent with the observations made by [18]. We cannot exclude that the lack of effect is due to the low levels of play. Nevertheless, due to the absence of these within treatment differences, we draw the conclusion that play seems to be sensitive to order effects and should therefore only be used as an indicator of a secure base in counter-balanced designs. We further add that using play at all as an indicator of a secure base, presupposes that the dogs perform it sufficiently often for a comparison between the amount of play in the presence of the familiar and unfamiliar person to be reliable.

There is a similar study to the one reported in this paper to validate the use of the ASSP in humans [5]. In that study one group of children participated in the ASSP together with their mothers and another group participated with an unfamiliar woman. It was found that 1 year-old children returned to play at the same rate after being reunited with their mother as after being reunited with the unfamiliar person (equivalent to our comparison of episode 5 between treatments). However, 2.5 year-old children played less when they were left by their mothers in the room compared to when left by the unfamiliar person, indicating a greater effect of being separated from their mother. The older children however, played equally as much when either the mother or the unfamiliar person was present in the room in the two conditions. In dogs, the level of play behaviour during the SSP has also been reported to be influenced by age [17] although age effects were not the main focus of that study.

Given these results on the effect of age and previous experience of social play, the effect of a familiar person on play behavior of a child or dog seems variable. For this reason play does not seem to function as a clear indicator of whether or not the attachment figure is acting as a secure base during SSP studies in dogs.

### Proximity seeking behaviour

According to the attachment theory, attached individuals should want to be close to, or at least be oriented more towards, the attachment figure or where it has been [7], [10]. In this study, we found significant effects on the level of physical contact. Dogs initiated more physical contact with the familiar person than with the stranger in treatment FS while showing no preference for physical contact with either stranger A or B in treatment SS. Dogs also initiated more physical contact with the familiar person than with stranger A when comparing the same episodes across treatments. These results indicated that dogs did discriminate between the familiar person and the strangers and that dogs clearly preferred to be near the former.

This is in line with conclusions drawn by other authors [6], [13], [15], [21] and with the structure observed in child-parent attachment. It can therefore be concluded that the research dogs in this study were attached to a familiar person, in a similar way as has been shown in previous studies for dogs and their owners. Although perhaps not surprising to those working with research dogs, to our knowledge this is the first time it has been shown that there are indicators of an attachment bond between dogs in a laboratory setting and their caretakers.

### Greeting behaviour

One of the most important features to look at when assessing attachment is the behaviour of the attached individual upon reunion with its attachment figure [11]. The immediate reaction (measured during the first minute after reunion) to the familiar person or the strangers differed with regards to the amount of physical contact initiated by the dog, as discussed in the previous section. Such differences reliably indicate more comfort seeking behaviour by the dog towards the familiar person. On the other hand, that lip licking was higher during the first minute of episode 5 than it was in the first minute of episode 6 (in both treatments) can probably be explained by the fact that the dogs were completely alone prior to the reunion in episode 5, but already had human company prior to the reunion in episode 6. Lip licking is therefore probably a general response to the return of any person after being left alone [22].

In summary, all previous studies where the SSP has been used to measure dog attachment found a more intensive greeting response towards a familiar person compared to an unfamiliar person (e.g. [6], [15], [21], [23]) whereas effects on exploration and play behaviour are variable and context specific. This supports the views of several authors who have underlined that the response to reunion reveals more about attachment than does behaviour during separation [1], [24], [25].

### Heart rate responses

Mean HR decreased over time in both treatments, which is consistent with the order effects observed for the other measures. In this study there was no support from comparisons within or across treatments, that HR was affected by the presence of the familiar person as HR changes could be explained by physical activity or by the reaction to the novelty when first entering the room. While [17] did not find any effects on HR of the accompanying person in the SSP, [26] did find some evidence that HR increased in the absence of their owners. In [16], an increase in HR was interpreted as an emotional arousal caused by being left alone. An increased HR observed when the stranger returned was suggested to be linked either to a negative reaction of fear or defence, or to a positive arousal (‘any’ company is preferable to being alone). The low levels of wariness and high amount of approaching behaviour towards strangers at reunion in general support the latter.

### A critical evaluation of the use of SSP in dog-human studies

Due to inconclusive results regarding the secure base effects on the level of exploration and play in the presence of the owner, it has been questioned whether or not there really is an attachment bond between the dog and its owner [6]. Attachment, however, is more than an individual's sense of security [27]. From human psychology even if a child is not considered to be securely attached (i.e. showing no signs of the parent acting a secure base), they are not necessarily regarded as being less attached to the parent [9], [24], [25], but merely they have another style of attachment. Considering the evolutionary aspects of attachment, there is unlikely to be only one type of attachment style that is adaptive, since which is the most appropriate style will depend on variations in specific environments or niches [25], [28]. Given that there are a variety of attachment styles among infants, we propose that more focus should be on different attachment styles in future studies. Because there are different attachment styles, which seem to have been partly ignored in earlier discussions when assessing the attachment between dogs and humans, the lack of evidence of a secure base effect on exploration or play does not mean that there is no attachment to the human, but rather that the type of attachment may not be the secure type.

So we must ask ourselves, is the SSP a reliable method to use when assessing the type of attachment in dogs? In human psychology, the ASSP has been criticized due to the very strict procedure and context, the unnatural situations included in the test and because it is only based on stress relief and reassurance in the face of novelty and separation (e.g. [27], [29]). The assessment is usually performed during a sensitive period of child development where the child reacts with a stress response towards a stranger, whereas it is performed on dogs of all ages. If properly socialised, dogs are probably more used to unfamiliar people than is a young child and also more used to being separated from their attachment figure (their owner). During the SSP, dogs generally show little wariness towards the stranger (e.g. [18]) and most dogs approach and greet the stranger at their first encounter [6], [15]. This is in contrast to what is commonly observed in young children, who initially stay close by their parent for comfort [5], [30]. Stranger acceptance starts to increase when the child reaches the age of 3 years, while proximity seeking behaviour towards the parent remains unchanged [31]. For this reason, the ASSP is considered to be an inappropriate method to study the more complex attachment behaviour in older children (>2 years old). Instead, observation of reunion between children and parents are commonly used (e.g. [24], [32]) and considered to be a good measure of attachment styles in children [9], [11]. It may be that more emphasis on reunion behaviour might be a fruitful line of research to investigate attachment in dogs. Moreover, due to the low levels of wariness towards a stranger during the SSP, an alternative ‘stressor’ to a stranger might be more challenging and so activate the attachment system in dogs.

According to the results in our study, order effects were evident in the SSP for exploration, one of the behaviours previously used as a measurement of a secure base in dogs. In contrast, proximity seeking behaviours (physical contact) and behaviours during greeting did not seem to be *as* affected by the order of episodes and are therefore probably better candidate behaviours when measuring attachment in dogs. Most studies show higher levels of physical contact with the owner during reunion in the SSP despite slightly different definitions and recording methods [6], [13], [15], [17], [18], [23]. Researchers therefore agree that dogs show a selective response to their attachment figure by interacting more intensively and/or for a longer duration with a familiar person than with a stranger at reunion, but the details of the interaction (i.e. the greeting behaviour itself) have not been investigated in the same way as within human psychology. A difference in greeting intensity however is not evidence of an attachment bond, without additional evidence of a difference in the type and quality of the interaction with the familiar person.

An important methodological difference between ASSP assessments in human relationships *vs*. the SSP dog attachment studies is that the former is completely score-based and has a slightly flexible procedure, while the latter uses quantitative measures in a very strict procedure to evaluate the relationship. In human studies, it is the dynamics of the interactive behaviour that is studied, such as the response to separation in relation to reunion behaviour, to classify the style of attachment. We propose that the sequence of behaviours shown upon reunion should be studied, such as approach, avoidance, extended duration of physical contact (resembling the ‘clinging’ behaviour observed in insecurely attached children), together with behaviours used in social contexts (such as tail wagging and lip licking). That is to say, studies of attachment in dogs should focus more on reunion behaviour, based on theories and methodology from human psychology, but these must be adapted to fit the dog-human relationship which means that further research is needed to find a suitable design, using a larger sample of dog-human dyads. In order to investigate the responsiveness of the attachment figure, which is assessed in human studies, we also propose that the behaviour of the owner should be recorded. In this way perhaps further investigations will also reveal different styles of attachment in dogs and these possible styles could then be linked to other features of dog and owner interactions. For example they may help understand mechanisms underlying conflicts in the dog-owner relationship, such as behavioural problems. Studies of attachment styles may also be a useful tool with which to explore the effect of selection of breeds and the effect of early socialization with humans. Thus despite criticisms of how it is currently used in dog-human interaction studies, there is clearly potential to use versions of the SSP in dog-human interaction studies. This study however, highlighted some of the previously discussed behaviours that could be sensitive to order effects and the results can hopefully highlight potential risks also in other versions of the SSP.

We used research dogs in our study who may not reflect the same responses as those shown by companion dogs living together with their owner, but as stated earlier the aim with this study was to investigate the SSP methodology. Although it is noteworthy that the results indicated dogs were attached to the familiar person. The number of animals included in the study was limited due to available subjects controlled for breed, sex and previous experience. Despite this, the analyses indicate that the number of animals was sufficient when dogs were used as their own controls. This may imply that even studies with access to a potentially unlimited number of pet dog-owner dyads could reduce the actual number of dyads used in their experiments, without compromising the statistical power of their study, if they use each dog as its own control. Regarding the methodology though, with hindsight it would have been interesting to include play invitations from the familiar person/stranger A, mainly to rule out that these dogs simply did not engage in social play with anyone. However, we still argue that play with the ‘owner’ should only be measured to reflect the effects of a possible secure base, and should not be compared to the level of play with the stranger. Excluding play invitations from the familiar person (or stranger A) also made these persons very passive throughout the test (except while greeting the dog) which may also have affected the dogs' general reactions to the test.

## Conclusion

The results from a controlled experiment with a homogenous group of research dogs, lead us to propose that the sequence in which the familiar person and the stranger appear with the dog in future SSP studies should always be counterbalanced to control for order effects in the test. We speculate that this slight modification would make it a more reliable test of attachment between companion dogs and their owners. We also propose that more emphasis is placed on the sequences of behaviour when the familiar person or the stranger and dog are reunited, since behaviour at this time seems to be a more robust indicator of the attachment bond. These sequences of behaviour could be compared either within a counterbalanced version of the SSP test or in a new test focusing more on separation and reunion in different contexts where the attachment system is challenged. Finally, we speculate that it would be interesting not only to study the secure style of attachment between dogs and humans, since it is unlikely that all dogs share the same style of attachment towards their owners. Increased knowledge about different attachment styles could shed light on the factors influencing the success of particular dog-human relationships.

## Supporting Information

Appendix S1 **Ethogram.** Ethogram and sampling methods.
(PDF)
