# Supplementary material for: Evaluating the Strange Situation Procedure (SSP) to Assess the Bond between Dogs and Humans
Source: PLoS One. 2013 Feb 20;8(2):e56938. doi: 10.1371/journal.pone.0056938 (PMC3577677; doi:10.1371/journal.pone.0056938)
Supplement: Appendix S1 — Ethogram. Ethogram and sampling methods. (PDF) [file pone.0056938.s001.pdf]

Appendix S1. **Ethogram**. Ethogram and sampling methods

BEHAVIOUR	DEFINITION	Sampling method
**Main behaviour**		
Lying alert	Lying down without its head in contact with the floor	Instantaneous (5s)
Lying resting	Lying down with its head in contact with the floor	Instantaneous (5s)
Sitting	Sitting up with front legs extended and hind legs curved	Instantaneous (5s)
Standing	Standing up with all four paws in contact with the floor/other object	Instantaneous (5s)
Walking	Walking around, moving	Instantaneous (5s)
Running	Running around, trotting or galloping	Instantaneous (5s)
**Secure base effects**		
Exploration	Motor activity directed towards any physical aspect of the environment, dog is sniffing/licking/manipulating something (not toys/leash/water bowl)	1/0 (5s)
Social play	Any motor activity directed towards a toy, with physical contact (chewing/biting/shaking from side to side/holding it in its mouth/scratching/batting with paw), chasing toy in movement (thrown by the human) when interacting with the stranger	1/0 (5s)
Individual play	Any motor activity directed towards a toy/leash/water bowl (or other object), including chewing/biting/shaking from side to side/holding it in its mouth/scratching/batting with paw/tossing WITHOUT any interactions with a human	1/0 (5s)
Located in Room 2	> 50% of the dog's body is located in room 2	1/0 (5s)
**Proximity seeking behaviours**		
Located in familiar person's zone	> 50% of the dog's body is located in the owner's zone	Instantaneous (5s)
Located in stranger's zone	> 50% of the dog's body is located in the stranger's zone	Instantaneous (5s)
Located near entrance door	> 50% of the dog's body is located in the zone closest to the entrance door	Instantaneous (5s)
Oriented towards familiar person	The dog is sitting, standing or lying and focused on (muzzle directed towards) the familiar person for > 2s	1/0 (5s)
Oriented towards stranger	The dog is sitting, standing or lying and focused on (muzzle directed towards) the stranger for > 2s	1/0 (5s)
Oriented towards door	The dog is sitting, standing or lying and focused on (muzzle directed towards) the entrance door for > 2s	1/0 (5s)
Following familiar person	Locomotion in the same direction as the familiar person, who is also in locomotion	1/0 (5s)
Following stranger	Locomotion in the same direction as the stranger, who is also in locomotion	1/0 (5s)
Physical contact familiar person	Physical contact with familiar person, initiated by the dog, regardless of visual orientation (leans/jumps up on/nudges/licking)	1/0 (5s)
Physical contact stranger	Physical contact with stranger, initiated by the dog, regardless of visual orientation (leans/jumps up on/nudges/licking)	1/0 (5s)
Physical contact door	Physical contact with entrance door, regardless of visual orientation (leans/jumps up on/nudges/licking)	1/0 (5s)

Vocalisation	Whining, barking, growling, howling	1/0 (5s)
**Greeting behaviour**		
Lip licking	Dog is snout licking, tongue visible	Continuous
Tail wagging	Repetitive wagging movement of the tail	1/0 (5s)
Body shaking	Dog shakes any part of or the whole body from side to side	1/0 (5s)
Body stretching	Dog is extending/stretching part of or whole body	1/0 (5s)
Yawning	Dog opens its mouth widely and inhales	1/0 (5s)
